# Automatic determination of 3D orientations of fossilized oyster shells from a densely packed Miocene shell bed

**DOI:** 10.1007/s00531-018-1591-0

**Published:** 2018-02-23

**Authors:** Ana Puttonen, Mathias Harzhauser, Eetu Puttonen, Oleg Mandic, Balázs Székely, Gábor Molnár, Norbert Pfeifer

**Affiliations:** 1 Department of Geodesy and Geoinformation, Vienna University of Technology, Vienna, Austria; 2 Geological Paleontological Department, Natural History Museum Vienna, Vienna, Austria; 3 Department of Remote Sensing and Photogrammetry, Finnish Geospatial Research Institute in the National Land Survey of Finland, Masala, Finland; 4 Centre of Excellence in Laser Scanning Research, Masala, Finland; 5 Department of Geophysics and Space Science, Eötvös Loránd University, Budapest, Hungary

**Keywords:** Terrestrial laser scanning (TLS), 3D-point cloud, Oysters, 3D orientations, Sedimentology, Digital documentation

## Abstract

Shell beds represent a useful source of information on various physical processes that cause the depositional condition. We present an automated method to calculate the 3D orientations of a large number of elongate and platy objects (fossilized oyster shells) on a sedimentary bedding plane, developed to support the interpretation of possible depositional patterns, imbrications, or impact of local faults. The study focusses on more than 1900 fossil oyster shells exposed in a densely packed Miocene shell bed. 3D data were acquired by terrestrial laser scanning on an area of 459 m^2^ with a resolution of 1 mm. Bivalve shells were manually defined as 3D-point clouds of a digital surface model and stored in an ArcGIS database. An individual shell coordinate system (ISCS) was virtually embedded into each shell and its orientation was determined relative to the coordinate system of the entire, tectonically tilted shell bed. Orientation is described by the rotation angles roll, pitch, and yaw in a Cartesian coordinate system. This method allows an efficient measurement and analysis of the orientation of thousands of specimens and is a major advantage compared to the traditional 2D approach, which measures only the azimuth (yaw) angles. The resulting data can variously be utilized for taphonomic analyses and the reconstruction of prevailing hydrodynamic regimes and depositional environments. For the first time, the influence of possible post-sedimentary vertical displacements can be quantified with high accuracy. Here, the effect of nearby fault lines—present in the reef—was tested on strongly tilted oyster shells, but it was found out that the fault lines did not have a statistically significant effect on the large tilt angles. Aside from the high reproducibility, a further advantage of the method is its non-destructive nature, which is especially suitable for geoparks and protected sites such as the studied shell bed.

**Electronic supplementary material:**

The online version of this article (10.1007/s00531-018-1591-0) contains supplementary material, which is available to authorized users.

## Introduction

To improve our understanding of hydrodynamics from the past, it is important to study the bivalve shells adapted to specific local environments (Dunca et al. 2009; Versteegh et al. 2011). Consequently, shell beds may provide information on various physical processes that caused the accumulation. Many shell beds pass through a complex history of formation being shaped by more than one factor under different environmental conditions (Kidwell 1986, 1991; Fürsich and Oschmann 1993; Mandic et al. 2004; Zuschin et al. 2005). The shell orientation is one of the sources that can be used to reconstruct information about events and processes from the past. The orientation of objects on bedding planes is a central topic in sedimentology. The pioneering paper by Nagle (1967) experimentally tested the influence of waves and currents on shell orientation. The position of elongated objects, such as mollusc shells, was investigated in numerous papers, where the measurements aim at deciphering past depositional environments and hydrodynamic regimes, such as prevalent currents, wave action, storm directions, and turbidite flows (e.g., Grant et al. 1992; Hladil et al. 1996; Newell et al. 2007; Roberts et al. 2008; Harzhauser et al. 2015). In most cases, information on fossil orientations is only available from traditional field measurements, which are difficult to resample and sensitive to subjective interpretations. Manual measurements are also time-consuming, which limits the number of measurements taken, thus leading to spatially limited sampling. While direct field measurements are still common practice, already Brenchley and Newall (1970), improved the level of objectivity and reproducibility by deriving measurements from field photographs. A further drawback of most analyses is the 2D nature of the data, which focus only on the azimuth angle of the orientation. This angle is interpreted as an indicator of flow or transport direction, while having two more orientation angles from the shells can be an important source of information to report in more detail about depositional condition of the specimens. The advantage of 3D data is spatial positioning of objects, parallel or tilted to the bedding plane, which might reveal imbrication or can support finding object movements due currents, transport mechanisms, local fault lines, or encrustations. Lukeneder et al. (2014) and Mayrhofer et al. (2016) developed a 3D approach to capture the orientations of ammonite shells in a sediment block by virtual shell reconstructions, derived from serial grinding techniques. The resulting 3D visualizations allowed measuring the azimuth angle and two more angles. The obvious disadvantage of this approach is its destructive nature, resulting in the complete loss of the studied objects.

A major advantage and potential is offered by the application of the newest technology, terrestrial laser scanning (TLS), and close range photogrammetry, which provides millimeter-level measurement accuracy and allows rapid acquisition of huge data sets (Stylianidis and Remondino 2016). However, only few studies so far have tried to take advantage from TLS for sedimentological studies. Millane et al. (2006) used laser scan-derived digital elevation models to quantify imbrication of pebbles and to reconstruct flow directions in alluvial deposits. Sediment transport processes were also the focus of Hodge et al. (2009), who used TLS to identify grain-scale topographic data from gravel-bed surfaces. Heritage and Milan 2009 and Smith et al. (2012) applied TLS to determine the shape, orientation, and grain roughness in gravel-bed rivers. A TLS-derived topographic model of a fluvial system was the base for Brasington et al. (2012) to test hydrodynamic simulations and quantify erosion and transport rates. Similarly, Milan et al. (2007) used 3D laser scanning to quantify small-scale changes in river channels to assess erosion and deposition volumes. Thus, most applications focused on fluvial sedimentation, whereas the advantages of TLS were rarely exploited for paleontological data sets. An exception is the papers by Harzhauser et al. (2015, 2016), who used TLS-derived data transferred into a GIS database to quantify and assess taxonomic composition, size distribution, degree of fragmentation, and 2D orientation of oyster shells in a vast Miocene shell bed. The same data set, accessible via PANGAEA (Djuricic et al. 2016b), is utilized herein, to develop a new approach to assess data on 3D orientation of elongated objects in geoscience by applying non-destructive, high-resolution TLS. Hence, this study aims to automatically derive the three angles of orientation of an oyster shell to better explain transport direction or presence of imbrication. For this, we develop a method to calculate the angles of rotation from the 3D-point cloud data representing the oyster shell.

## Geological setting

**Fig. 1:**
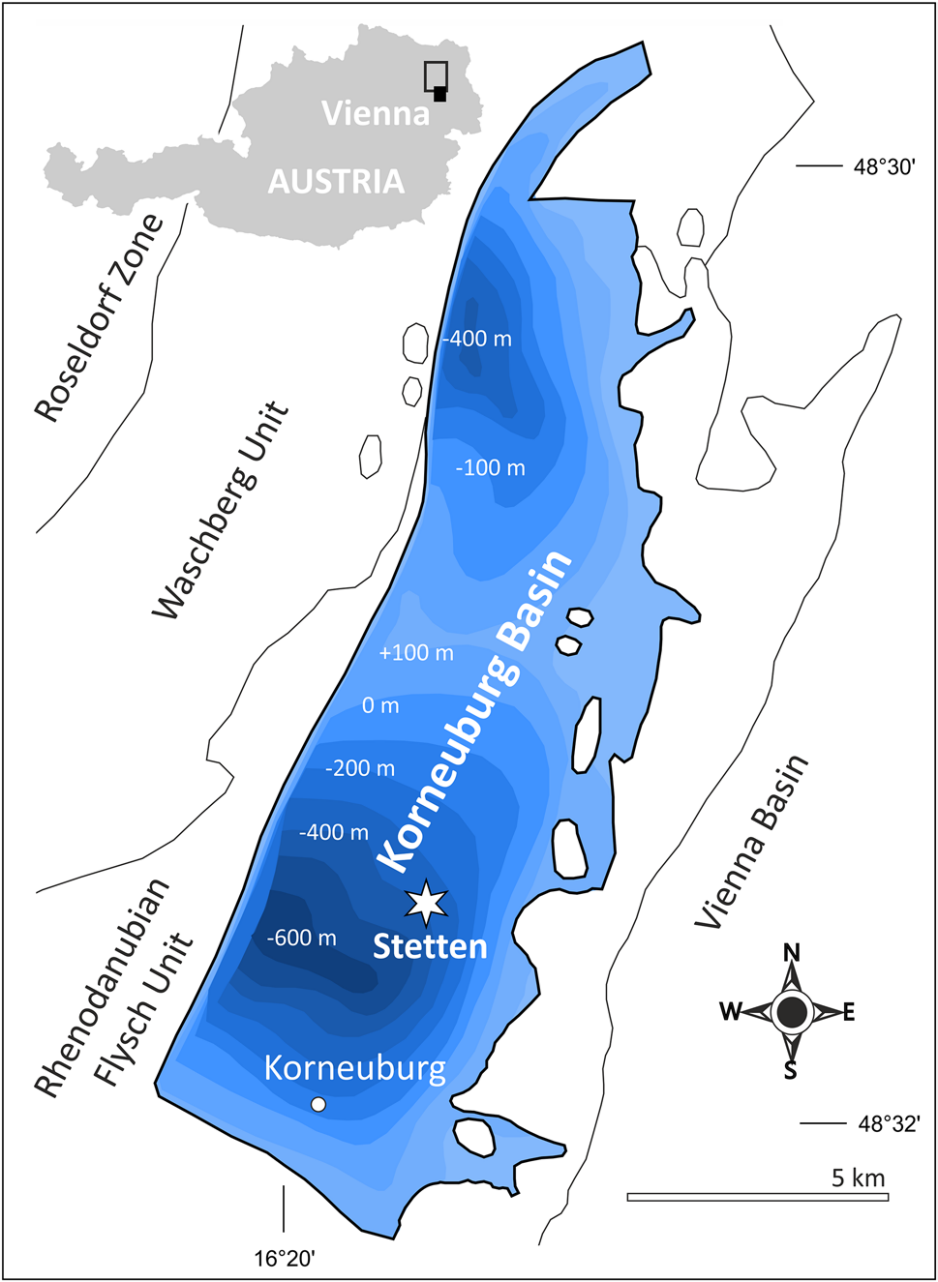
Geographic map from 16.5 Ma ago (Zuschin et al. 2014), at Stetten site (48°22′03.33N, 16°21′33.22E) as a part of the small Austrian Korneuburg Basin in Lower Austria

The studied site at Stetten (48°22′03.33N, 16°21′33.22E) in eastern Austria is part of the about 20-km-long and about 7-km-wide Korneuburg Basin, which is a lower Miocene halfgraben within the Alpine–Carpathian thrust belt. The basin fill comprises the about 600-m-thick siliciclastic Korneuburg Formation of Burdigalian (= Karpatian) age, which is tilted ca. 25° in western direction. The succession reflects a rapid shift from estuarine and shallow marine depositional environments with repeated formation of coastal swamps around 16.5 Ma ago (Zuschin et al. 2014—Fig. 1).

The protected site is part of the geopark “Fossilienwelt Weinviertel” and exposes the world’s largest fossil oyster biostrome which was excavated during field campaigns of the Natural History Museum Vienna between 2005 and 2008. The shell bed is a single, about 15–25-cm-thick horizon intercalated in coarse, poorly sorted sand with scattered plant debris and mudclasts. It is interpreted to have formed in a subtropical estuary as an event layer initiated by a major storm or tsunami (Harzhauser et al. 2015, 2016). The excavated and exposed shell accumulation now covers an area of 27 × 17 m (459 m^2^) comprising about 54,000 shells of the giant oyster *Magallana gryphoides* (Schlotheim, 1813) and is presented to the visitors of the geopark in the so-called “oyster hall”.

Note that this species was formerly placed in the genus *Crassostrea* (Sacco, 1897). Recently, molecular data suggested that the extant Indo-West Pacific (IWP) representatives of this taxon form a distinct clade for which the genus *Magallana* was established by Salvi and Mariottini (2016). As the Miocene species belongs to this post-Tethyan IWP clade, we place it here in *Magallana*.

## Methods

**Fig. 2:**
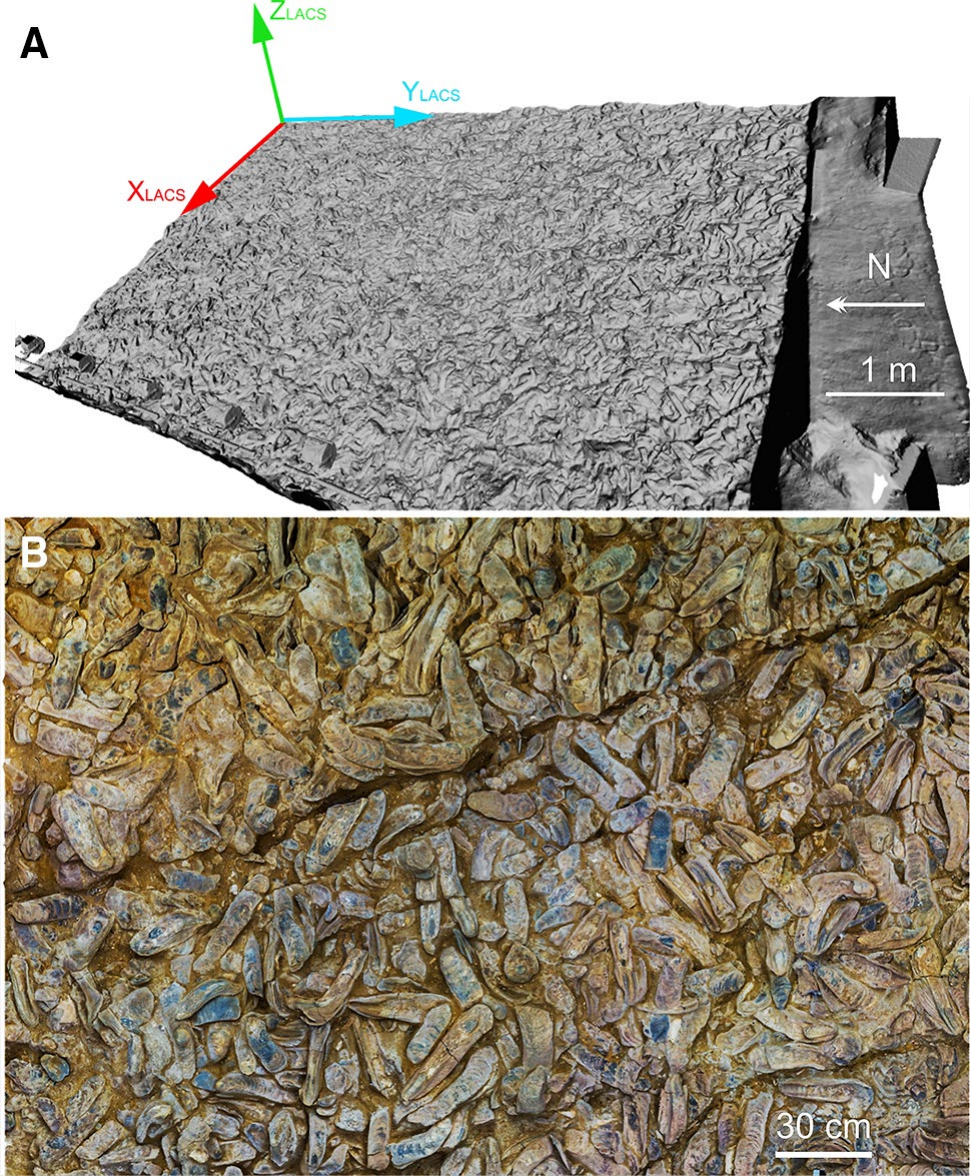
**a** Digital surface model of the shell bed (LACS is the reference coordinate system of the surface). **b** Orthophoto (2 m $\times$ 3 m) showing the excavated surface of the shell bed and a fault

Terrestrial laser scanning was used to document the shell bed as a georeferenced 3D-point cloud. In addition, 300 images with a resolution of 0.6 mm were acquired for the texture information. The derived digital surface model (DSM) forms a rectangle of 459 m^2^. An orthogonal regression plane was determined from a subset of the TLS point cloud. This subset was a subsampled point cloud that ensured that the global approach (working on the entire shell bed at once) was successful by avoiding numerical problems (covariance matrix of one billion points). The plane was determined by a principal component analysis (PCA) of the point cloud. From this, the rotation matrix to transform the points from the acquisition coordinate system into the local analysis coordinate system was determined (see Djuricic et al. 2016a for technical details). The entire area was rotated by 20.26° to define a local analysis coordinate system, the LACS. Its axes follow the dip and strike directions; the third axis, *Z*, is orthogonal to the adjusting plane through the field. Axis *X* extends downward in the field (west direction), and *Y* is horizontal pointing to the south (Fig. 2). Thus, the *Y*-axis is approximately parallel to the length axis of the oyster hall with a deviation of 3°. The definition of the LACS is described in detail by Djuricic et al. (2016a, b) as well as the transformation to the Universal Transverse Mercator (UTM) System to allow comparison with other geological data from the region.

### Technical setup

The excavated shell bed (27 m × 17 m) is protected by an indoor hall as part of the geotainment park, “Fossilienwelt Weinviertel”. The low illumination conditions and limited access were a big challenge for the technical setup. It required contactless access across the reef. Control points were distributed on the reef in a grid of 6 m × 3 m and on the visitors’ platform around the reef (Djuricic et al. 2016a). They served the purpose of registering 3D laser scans, as well as being a link to an external coordinate system, i.e., Universal Transverse Mercator (UTM). Their precision is better than 2 mm. The geometrical data were generated with a high-speed 3D laser scanner FARO Focus3D, resulting in a point cloud with a resolution of 1 mm. The texture of the shell bed was captured with a Canon 60D camera with a Canon EF 20 mm f2.8 lens, resulting in a resolution of 0.6 mm. There is no possibility of positioning an instrument on the oyster reef because of conservation concerns, but a long mobile bridge was constructed, and most of the data were acquired from it using a stiff and heavy metal structure built to carry the scanner. A special light tent was constructed, including the camera on the top, illuminated simultaneously from several sides with studio spots to achieve a homogeneous illumination over the entire reef (Djuricic et al. 2016a).

The laser scanner that was remotely controlled collects 3D points with a rate of up to 1 MHz and a range of up to 100 m with a 3.8-mm-diameter laser beam (FARO 2017). Experimental analysis has shown that, over short distances, the single-point measurement precision of FARO terrestrial laser scanning data is actually ~ 2 mm up to 10 m range (FARO 2017). To increase the precision of the result, i.e., the surface model of the reef, we applied point-based filtering (i.e., averaging). Occlusions were minimized by the setup of the device during scanning (high overlap and, therefore, various incidence angles). For the photogrammetric data acquisition, a uniform illumination was necessary to enable an image acquisition that was almost shadow free. Digital camera captured images, each 5184 × 3456 pixels, were mounted approximately orthogonal to the oyster reef plane. On average, the ground sampling distance (GSD) was 0.6 mm, and the footprint of the image was ~ 3.1 m × 2.1 m on the reef.

Scanning the fossil shell bed required multiple viewpoints to ensure minimization of scan shadows and to provide homogeneous point coverage. The scanning height of 1.5 m above the reef also led to the capturing of overhangs. Thus, scans were acquired in a regular grid of 2 m × 2 m, with the distance between the laser scanning points ranging from 0.7 mm (center) to 1 mm (outer edges) per scan. In total, 83 scan positions were sufficient to collect data covering the entire reef. From all those positions, approximately one billion points were acquired at the site, corresponding to ~ 150 points per square centimeter. The high number of nearly orthogonal scanning positions enabled a homogenous point density on the surface of the oyster reef. In each scan, 15 or more control points (i.e., planar photogrammetric targets) were identified, covering the entire field of view. A global accuracy of better than 3 mm was achieved, but local discrepancies between overlapping scans are smaller, in the region of a maximum of 1–2 mm. This means that local precision (std. dev.) is in the order of 1 mm.

Photogrammetric images were oriented by means of a bundle block adjustment (BBA) with control points (photogrammetric targets and distinct points in the terrestrial laser scanning data) on the reef. We used Agisoft PhotoScan (PhotoScan software, http://www.agisoft.com/) for automatic feature extraction, matching, and relative orientation of all images. Measurements of photogrammetric targets and distinct points were added manually. Subsequently, a datum transformation was applied with the control points. From the residuals in the BBA, using also distinct features in the point cloud, we estimated the global accuracy to be 3 mm.

We established the following coordinate systems to reconstruct the original situation during shell-bed formation for subsequent geological and paleontological analyses: (1) acquisition coordinate system (ACS); (2) local analysis coordinate system (LACS); and (3) Universal Transverse Mercator (UTM) system. The ACS is a local coordinate system used during the measurements with a total station and a TLS. The LACS is a coordinate system defined by a Euclidean transformation from the ACS. It represents the “horizontal geological” coordinate system. The axes follow the dip and strike directions, and the third axis, *Z*, is orthogonal to the adjusting plane through the field. Axis *X* extends downward in the field, and *Y* is horizontal. Transformation from the ACS to the UTM System was performed to ensure compatibility with future campaigns and to allow for comparison with other geological data from the region. The transformation parameters are given in Supplemental Table S2 (see PANGAEA, Djuricic et al. 2016b).

Data are stored tilewise, in total 81 tiles in a grid structure covering the entire shell bed (tile size: 2.1 m east–west and 3.1 m north–south). The area covered by shells and visible from the scan positions is 367 m^2^; the rest represents sandy area between shells (up to 459 m^2^).

### Orientation of the shells

**Fig. 3:**
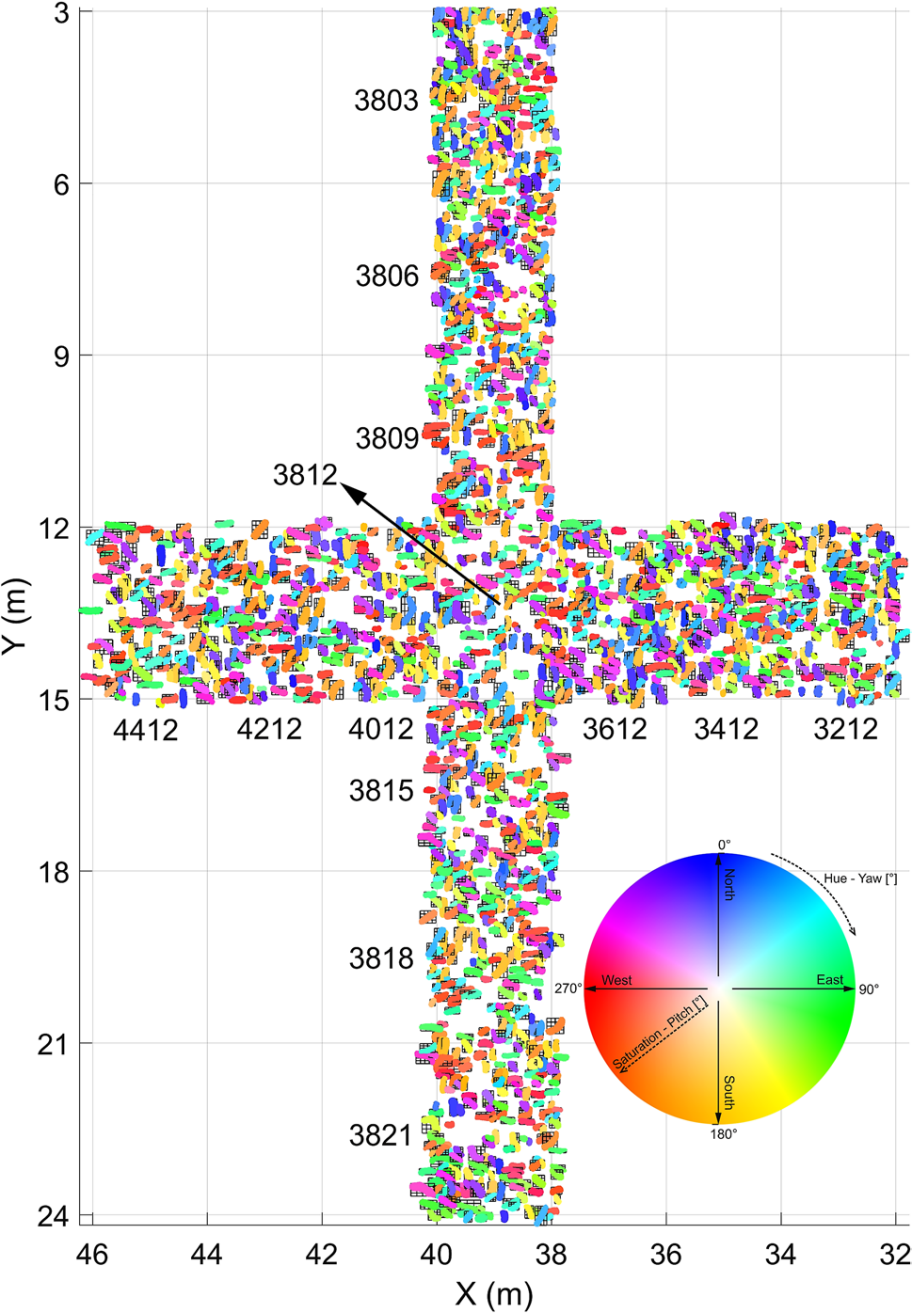
Shell orientation map for an N–S and a W–E transect with 1904 shells over 13 tiles (the same color wheel coding, as shown in Fig. 12)

The shells of *Magallana gryphoides* have an unusual big size (compared to present species). The largest specimen on the shell bed attains 602 mm in lengths and the mean length based on 1121 complete shells was calculated by Harzhauser et al. (2016) to be 237 mm. The boundary of each specimen (= shell margin) needs to be defined manually to assign the respective 3D points to the object. The high number of data points describing each specimen is a major advantage compared to the “three points per specimen”—approach as used by Mayrhofer et al. (2016). For practical reasons, two transects (N–S, W–E) were defined; each represented by 7 tiles of 2 × 3 m length, with the central one overlapping (in total 13 tiles, see Fig. 3). All objects within this area were manually outlined on the digital surface model and cross-checked based on the high-resolution orthophotos available online (PANGAEA, Djuricic et al. 2016b). In the two previous studies, all specimens were taxonomically identified (Harzhauser et al. 2015, 2016) and stored in a georeferenced ArcGIS database. Herein, we focus exclusively on 1904 shells of the oyster *Magallana gryphoides* defined in that database with attribute “complete” or “low fragmented”, i.e., having only minor damages that do not obscure natural shell shape. Moderately fragmented to strongly fragmented shells, representing less than half of the original length, are excluded as well as shells of other mollusc species. The reason for this selection is the elongate shape of the well preserved oysters, whereas most other objects lack a distinct main axis.

**Fig. 4:**
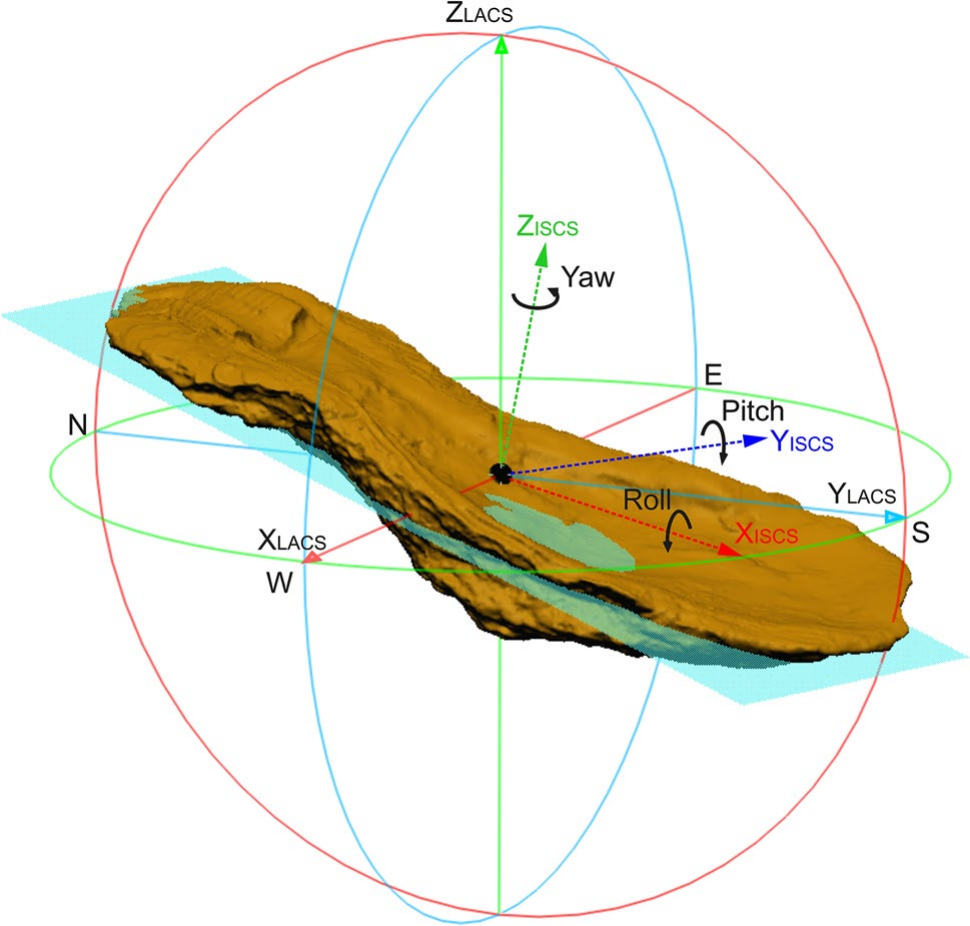
3D visualization of an elongated and tilted fossil oyster shell with the corresponding individual shell coordinate system (ISCS) and determined rotation directions in respect to the local analyses coordinate system (LACS). Note that positive direction of the *X*_ISCS_-axis is pointing from the position, where the hinge was located to the muscle position

**Fig. 5:**
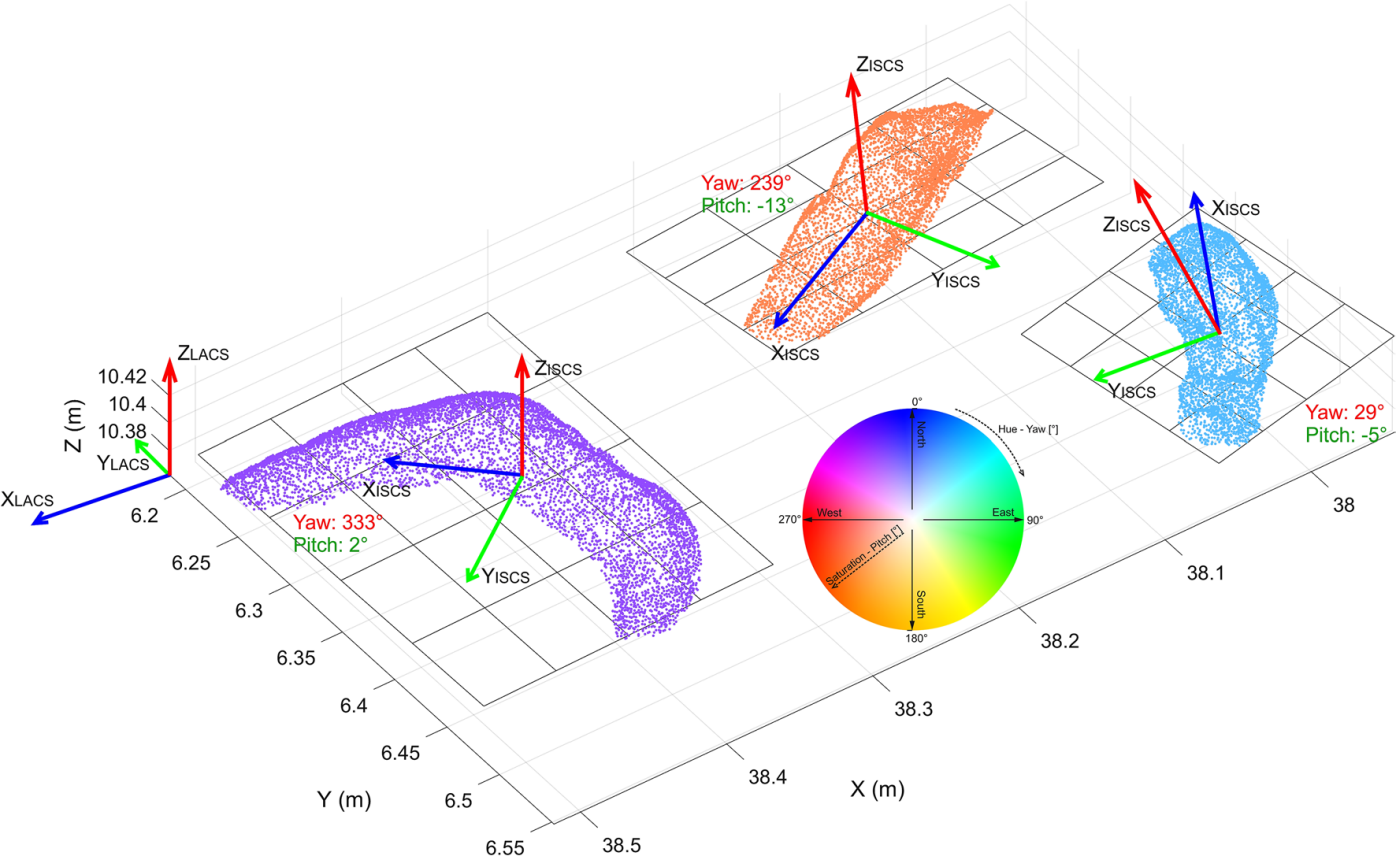
3D-point clouds of three individual shell examples and their rotations around *Z* and *Y* axes

For analyses, the oyster shells are treated as elongated flat elements in 3D space with predictable axes in all three spatial directions: shell length is larger than its width, which is larger than the height. Thus, the length, width, and height axes can be considered as pairwise orthogonal. These three axes are the base to measure rotation angles, following the principles originally used in aerial photogrammetry (Bäumker and Heimes 2002): rotation around yaw (= *γ*) (normal axis, *Z*), pitch (= *φ*) (lateral axis, *Y*), and roll (= *ρ*) (longitudinal axis, *X*). The yaw axis represents an axis drawn vertically in respect to the shell top surface, and it is perpendicular to the other two axes. The pitch axis is an axis running from the left to the right lateral sides of the shells (Fig. 4), corresponding to the width of the shell. The roll axis is defined as an axis drawn through the body of the shell from the anterior part (= hinge area) to the posterior shell margin, coinciding with the length of the shell (Figs. 4, 5). These axes are represented by the letters *X*_ISCS_, *Y*_ISCS,_ and *Z*_ISCS_ and the reference frame of the entire oyster reef is defined by the LACS, i.e., *X*_LACS_, *Y*_LACS,_ and *Z*_LACS_ axes.

We assume that the shells are evenly captured by data points on the exposed shell surface, which exhibits the extension in length and width, thus representing the two larger axes. Note that we define the direction of the axes here. In such a case, the direction of those two axes can be computed automatically, as well as the extent along each of those axes. The third axis is determined by augmenting the first two vectors to a right handed three-dimensional Cartesian coordinate system. We are, furthermore, assuming that the third axis is pointing upwards. With other words, the oyster shells are “lying” and not “standing”. This approach does not provide information if the length axis points from hinge to posterior margin or vice versa and does not distinguish between convex-side up and convex-side down positions (= if interior or exterior side of the shell is visible). However, such contextual attributes can be added to the point cloud of each shell and its axes.

Based on empiric data and direct observation, we can postulate that the objects studied are generally lying, approximately flat on the underlying surface, i.e., the shell bed. No chaotic orientation was observed. This means that the length axis coincides approximately with this plane. As start (hinge side) and end (muscle side) of the shell can be provided by paleontological experts, the direction of the length axis can be given in the range of 0°–360°. This introduces additionally to axis direction also axis orientation. The positive *X*_ISCS_ axis points from the hinge to the muscle (Fig. 4). This angle represents the yaw, heading, or azimuth. An azimuth is an angle measured clockwise. The 0° direction is pointing North, the 90° is pointing East, and the 180° direction is pointing South. The rotation around the lateral axis for a flat lying object is 0°. It can be positive and negative (see Supplementary Appendix). This angle represents the pitch. If it is positive, it means that the positive length axis is pointing upwards. Finally, the rotation along the length axis represents the roll. It can be positive or negative as well. It is positive if the *Y*-axis is rotated into the direction of the *Z*-axis (Fig. 4). The superior coordinate system is the LACS. It is attached to the shell bed, with the *X*-axis pointing downwards along the fall line, the *Y*-axis approximately to south (with a deviation of 3°), and *Z*-axis upwards (Fig. 2).

The predefined order of rotations around the axes is necessary to define unique rotation angles. The rotation of a shell is described by the sequential rotation around the three axes in the sequence *Z*_ISCS_-axis (height axis of the object with the yaw angle γ), *Y*_ISCS_-axis (width axis with pitch angle φ), and *X*_ISCS_-axis (length angle with roll ρ^1^):

$$\left( {\begin{array}{*{20}{c}} {\cos - \left( {90^\circ +\gamma } \right)} \\ {\begin{array}{*{20}{c}} {\sin - \left( {90^\circ +\gamma } \right)} \\ 0 \end{array}} \end{array}\begin{array}{*{20}{c}} { - \sin - \left( {90^\circ +\gamma } \right)} \\ {\begin{array}{*{20}{c}} {\cos - \left( {90^\circ +\gamma } \right)} \\ 0 \end{array}} \end{array}\begin{array}{*{20}{c}} 0 \\ {\begin{array}{*{20}{c}} 0 \\ 1 \end{array}} \end{array}} \right)\left( {\begin{array}{*{20}{c}} {\cos - \varphi } \\ {\begin{array}{*{20}{c}} 0 \\ { - \sin - \varphi } \end{array}} \end{array}\begin{array}{*{20}{c}} 0 \\ {\begin{array}{*{20}{c}} 1 \\ 0 \end{array}} \end{array}\begin{array}{*{20}{c}} {\sin - \varphi } \\ {\begin{array}{*{20}{c}} 0 \\ {\cos - \varphi } \end{array}} \end{array}} \right)\left( {\begin{array}{*{20}{c}} 1 \\ {\begin{array}{*{20}{c}} 0 \\ 0 \end{array}} \end{array}\begin{array}{*{20}{c}} 0 \\ {\begin{array}{*{20}{c}} {\cos \rho } \\ {\sin \rho } \end{array}} \end{array}\begin{array}{*{20}{c}} 0 \\ {\begin{array}{*{20}{c}} { - \sin \rho } \\ {\cos \rho } \end{array}} \end{array}} \right).$$

The point coordinates of the object are formulated as *P*i = (*x*_i_, *y*_i_, *z*_i_), *i* = 1,… *n*, where the points *p*_1_…*p*_*n*_ belong to the oyster. The structure tensor *T* has the second central moments of the point coordinates. It is decomposed into the eigenvalues *λ* with associated eigenvector *υ*:

$$T{\upsilon _j}={\lambda _j}{\upsilon _j}.$$

With sorted eigenvalues *λ*_1_ > *λ*_2_ > *λ*_3_ and associated eigenvalues, matrix A = (${\upsilon _{1,}}{\upsilon _{2,}}{\upsilon _3}$) can be constructed (see Supplementary Appendix), where the eigenvectors and the individual shell axes are related as ${\upsilon _1}=$ *X*_ISCS_, ${\upsilon _2}=$*Y*_ISCS_, and ${\upsilon _3}=$ *Z*_ISCS_. The direction of the axes may have to be inverted to follow the orientation as described above (see Supplementary Appendix, also for the computation of the angles from matrix A).

### Statistical tests

We tested if the angles follow expected distribution for a zero mean of the pitch and roll angle by utilizing Student’s *t* test, and a Kolmogorov–Smirnov test for the uniform distribution of the yaw angle. One-sample *t* test can be applied to detect spatial differences in the data of 13 tiles. These tests if the patterns observed for each of the tiles are significantly different from the overall pattern. In the statistical tests, the null hypothesis is evaluated by comparing the test statistic against a threshold, which depends on the significance level, e.g., 5%, of the test.

### Testing the effect of fault lines on orientation of the shells

**Fig. 6:**
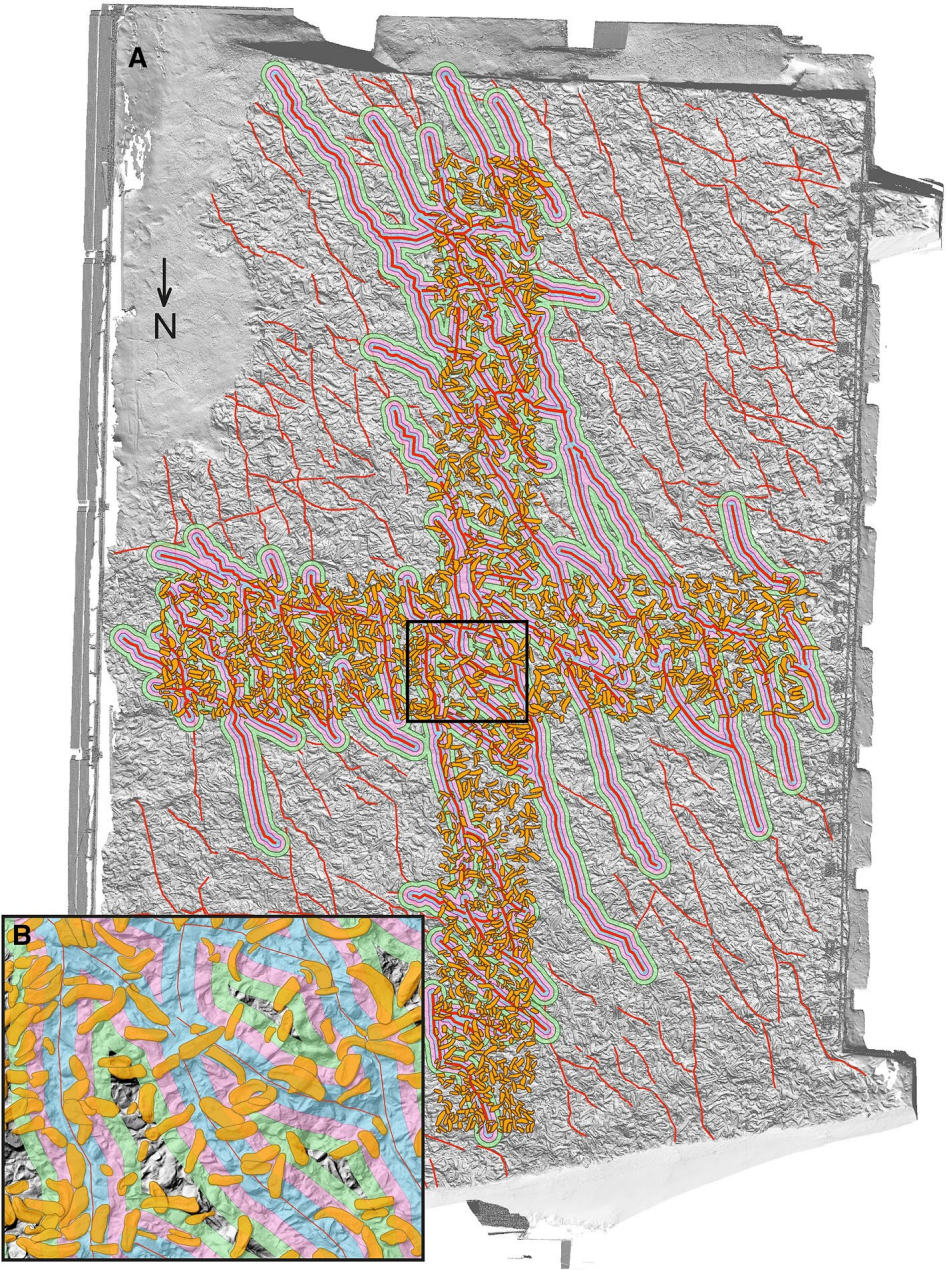
Individual shells within the multi-buffers surrounding the fault lines (red); buffer distances: blue: 0.1 m, pink: 0.2 m, green: 0.3 m. **b** Represents a magnification of a rectangle with a black outline in **a**

**Fig. 7:**
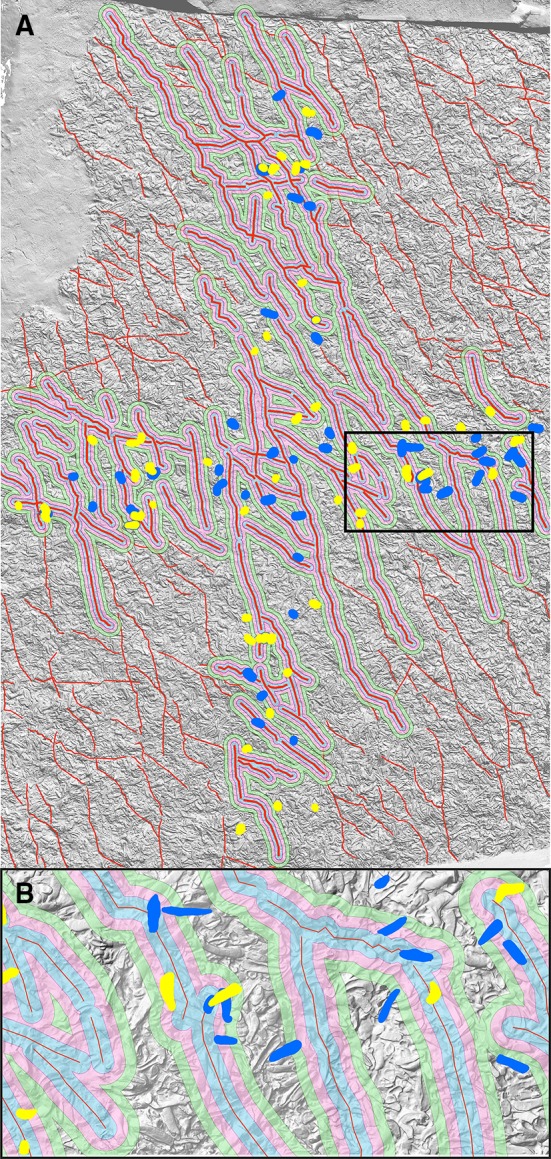
Shaded DSM overlapped with fault lines and strongly tilted shells: blue shells present roll angle tilt above 31.5° and yellow shells present pitch angle tilt above 18.4°. **b** Represents a magnification of a rectangle with a black outline in **a**

The NW–SE-trending fault system contains 318 identified faults ranging from 0.5 to 10 m in length, mapped using high-resolution digital surface models. They were provided as a manual reference acquired by a geological expert (Molnár et al. 2015). To see if the vicinity of fault lines influences the orientation of the shells, we calculated the shell distance from the nearest fault line using the multi-buffer method. It creates rings of equal distance around the reference fault lines. The output is a newly calculated field of nearest distances to the next fault line, which can be evaluated for any position but especially the closest distance of each oyster to its nearest fault line is extracted. Accumulative buffers are applied around 94 fault lines, mainly in the cross area (central part of the reef for which we have reference data). We took buffers of 0.01 and 0.05 m and three buffers of 0.1 m around the reference fault lines and checked their intersection with the oyster shell outlines (Table 2). These buffer distances were considered sufficient, because the 75th percentile of all oyster shell lengths in the used data set was 0.30 m, and the farthest distance of a shell from the nearest fault line was 0.83 m. The multi-buffer approach enables an accurate distance calculations by taking a total sum of the closest distances from extracted oysters to the reference fault lines (see Table 2 and Supplementary Table 4). Those shell-fault lines distances are displayed on the map (Figs. 6, 7) to show if the fault line vicinity and extreme tilt values of oyster shells are in statistical correlation. The correlation test should support the interpretation if some shell movement is related to tectonic activity and if the sandy sediment was still soft enough to allow for rotation of shells along the faults. The test inspects if rotations are significant or otherwise related to post-sedimentary displacements.

## Results

**Fig. 8:**
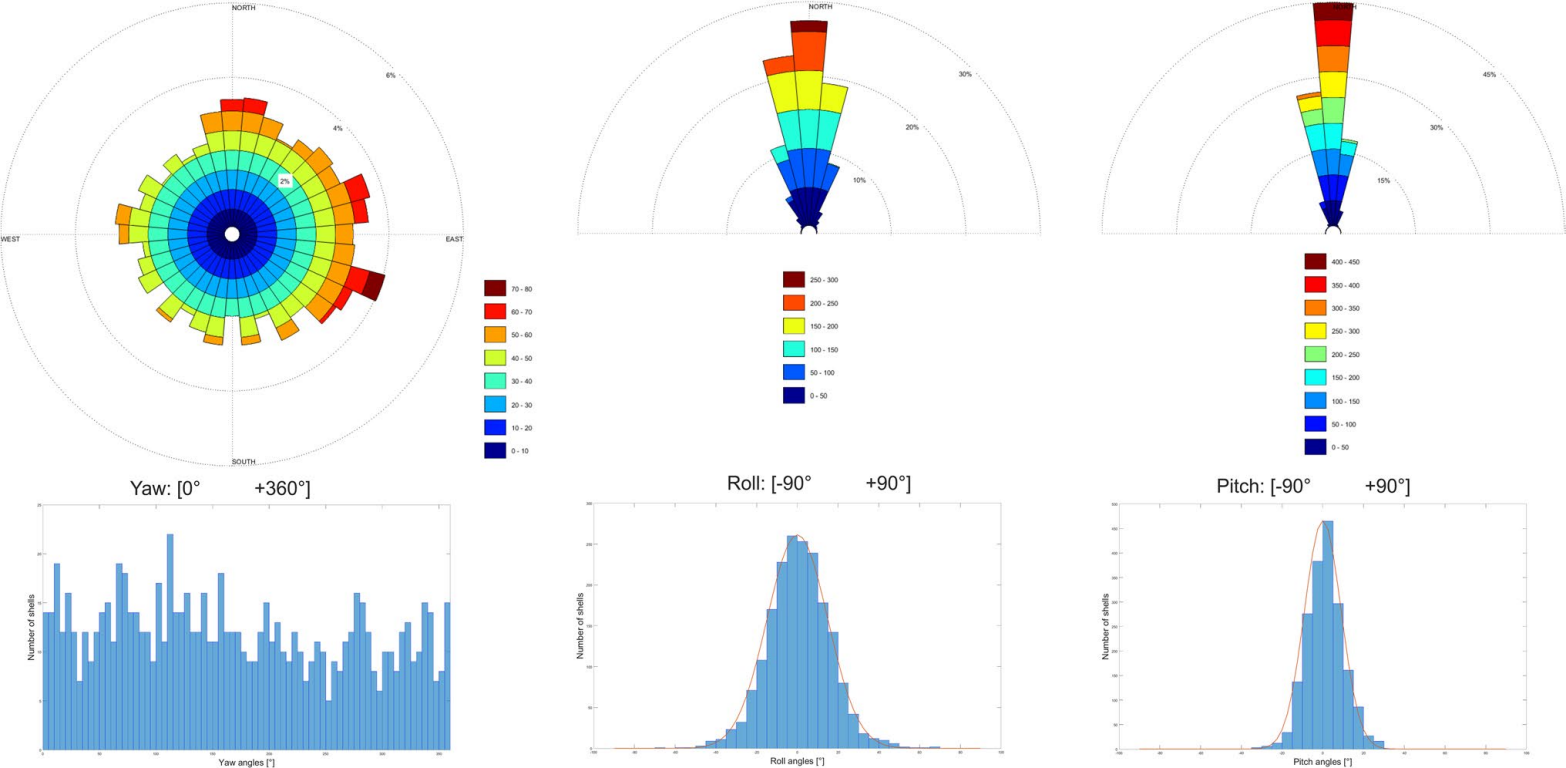
Rose diagrams and histograms based on *γ, φ*, and *ρ* angles of 1904 shells (bar width = 5°)

**Fig. 9:**
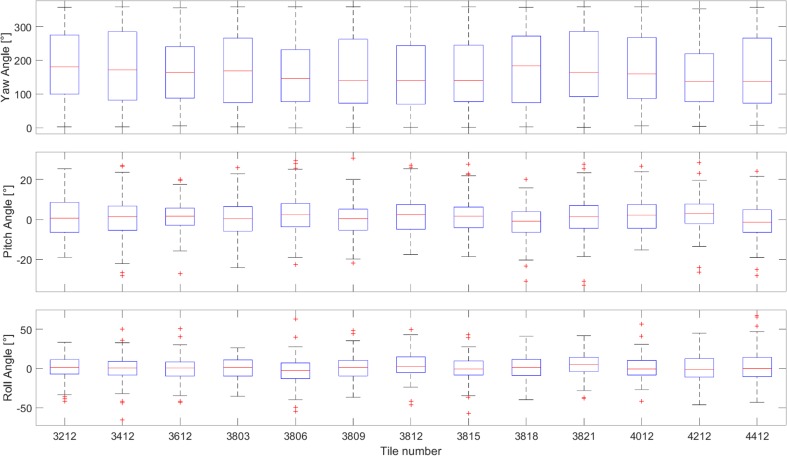
Tilewise distribution of yaw, pitch, and roll angles ranges in box plots

**Fig. 10:**
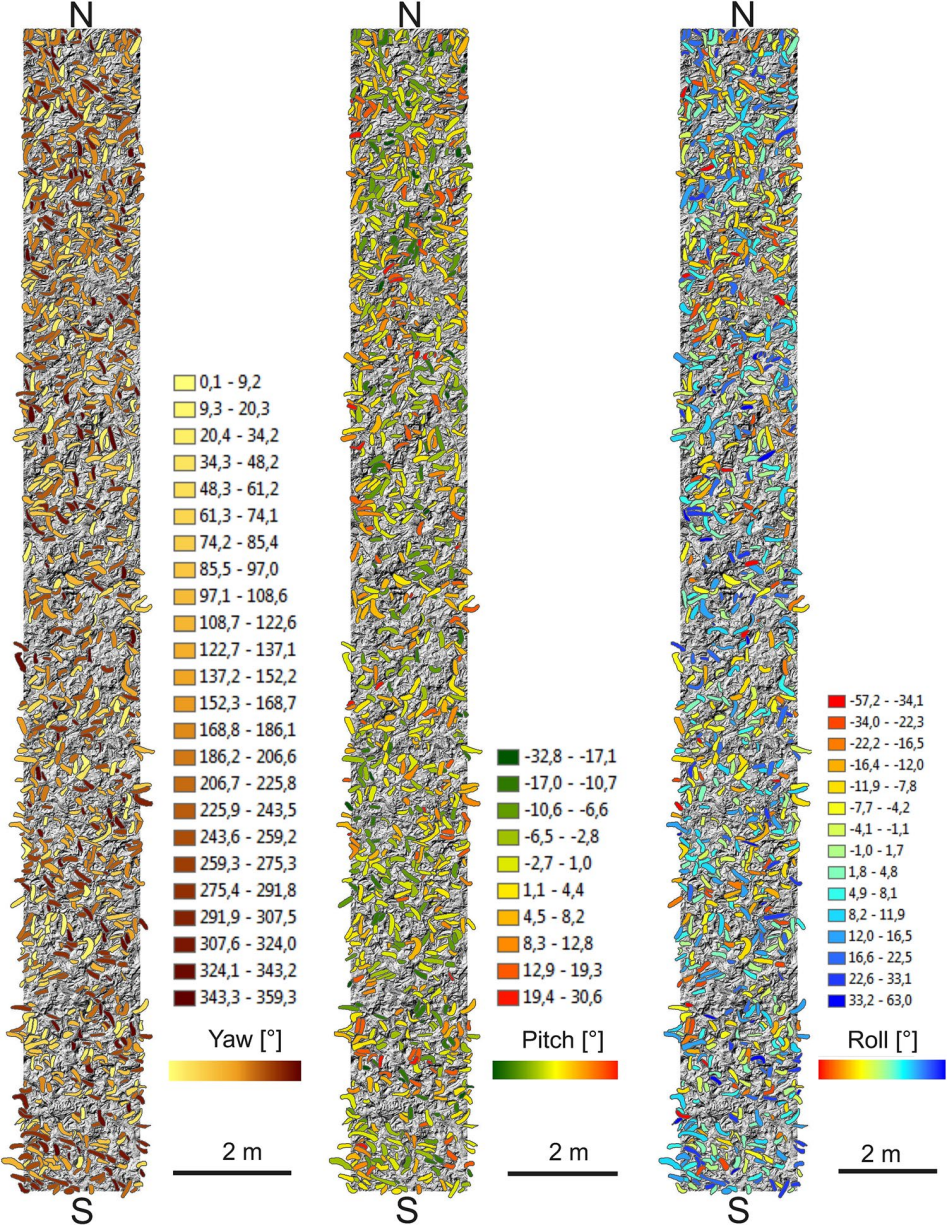

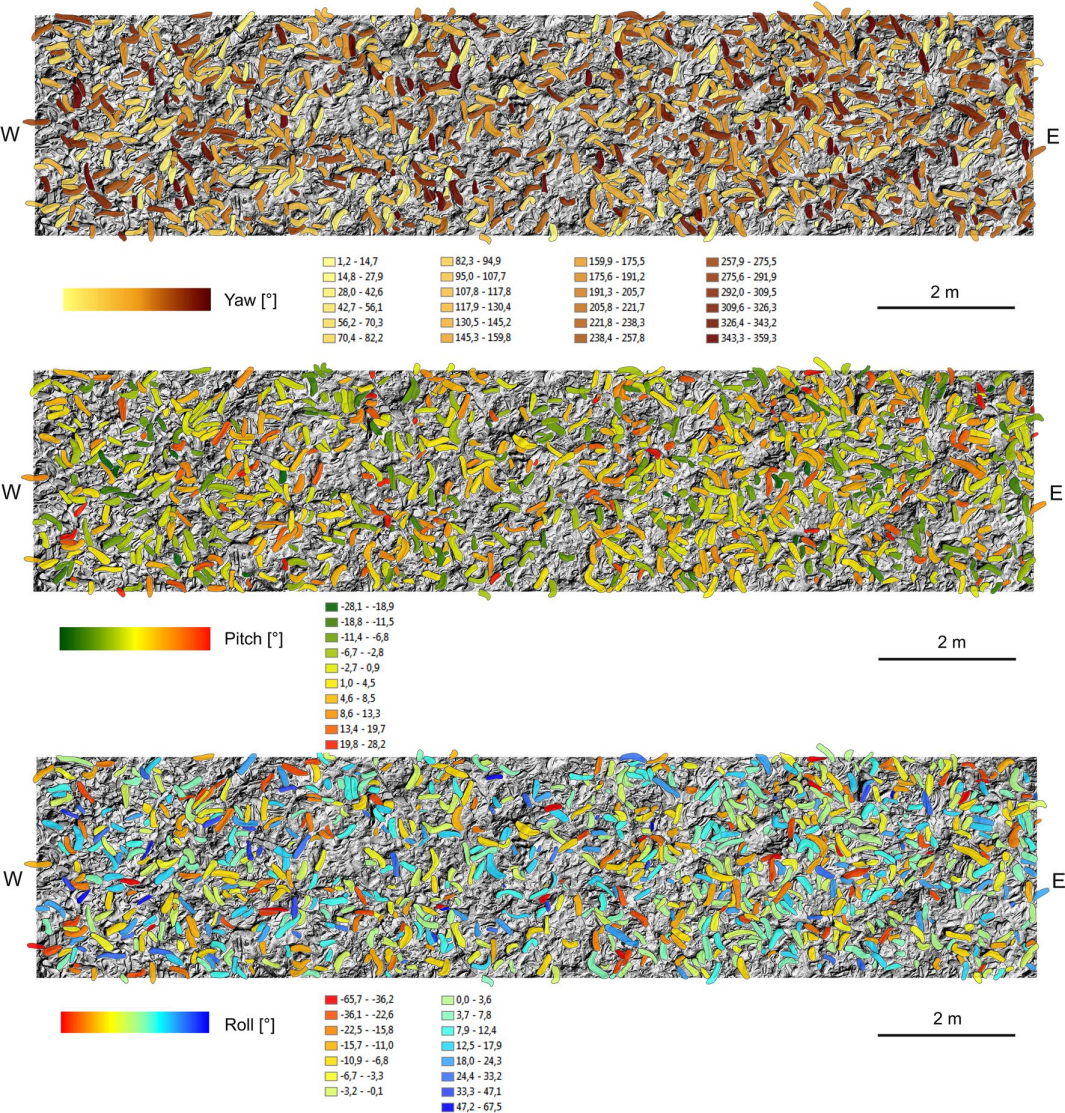
**a** N–S transect distribution of yaw, pitch and roll angles. **b** W–E transect distribution of yaw, pitch, and roll angles

**Fig. 11:**
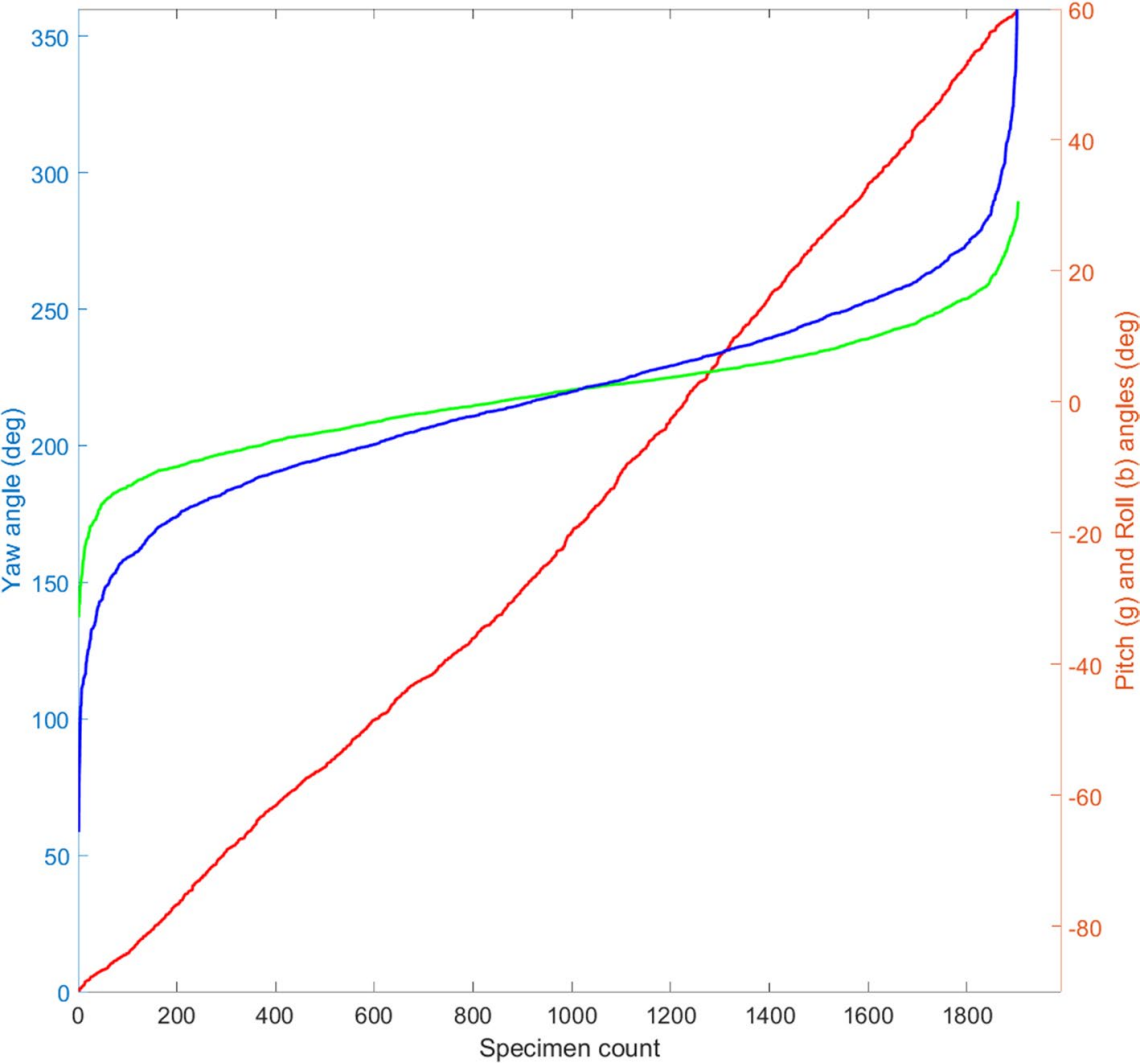
Yaw, pitch, and roll angles of 1904 oyster shells sorted in ascending order for each angle

Based on the manually determined shell polygons, yaw, pitch, and roll angles for 1904 shells were computed. The yaw angles represent a broad scatter in all directions, with a mean of 168.8°, and std. dev. 104°. The distribution in the interval [0°, 360°] has mean and std.dev. 180° and 104°, respectively. The distribution of the yaw angles is plotted in ascending order in Figs. 8, 9, 10a, b, 11. Figures evidently document the distribution closeness to the uniform distribution and, therefore, a highly random orientation pattern. The other two angles, pitch and roll, display distinctly less scattering. For the pitch angle (*φ*), a minimum value of − 32.8° and a maximum of 30.6° were observed (Table 1), with a mean of 1.11° (std. 9°). The roll angles* ρ* display a wider variation from − 65.7° to 67.5° with a mean of 0.74° (std. 15.4°). Distinct tails are formed in the distribution curves for pitch and roll angles, indicating the presence of few strongly tilted shells (Fig. 7).

**Table 1:** Student *t* test values (roll, pitch) for all shells and each tile

Tile number	Shells	*t*-roll	Roll (°) mean	Roll *h* (*p*)	*t*-pitch	Pitch (°) mean	Pitch *h* (*p*)
All	*n* = 1904	2.09	0.74	1 (0.04)	5.39	1.11	1 (0)
3212	*n* = 151	− 1.01	0.38	0 (0.76)	1.95	1.51	0 (0.62)
3412	*n* = 181	− 3.26	− 0.41	0 (0.32)	− 3.08	0.48	0 (0.38)
3612	*n* = 155	− 3.08	− 0.35	0 (0.34)	2.41	1.61	0 (0.42)
3803	*n* = 183	− 2.22	− 0.04	0 (0.46)	− 3.27	0.44	0 (0.31)
3806	*n* = 177	− 10.56	− 2.99	1 (0.002)	5.93	2.33	0 (0.09)
3809	*n* = 142	2.23	1.52	0 (0.55)	− 2.72	0.55	0 (0.44)
3812	*n* = 103	9.91	4.24	1 (0.03)	3.08	1.75	0 (0.47)
3815	*n* = 153	− 1.84	0.44	0 (0.79)	0.95	1.31	0 (0.77)
3818	*n* = 137	− 1.65	0.16	0 (0.64)	− 11.30	− 1.22	1 (0.00)
3821	*n* = 164	11.35	4.74	1 (0.00)	0.51	1.22	0 (0.89)
4012	*n* = 125	1.08	1.12	0 (0.76)	4.86	2.11	0 (0.21)
4212	*n* = 125	0.21	0.81	0 (0.96)	8.75	2.92	1 (0.02)
4412	*n* = 108	4.53	2.34	0 (0.40)	− 8.10	− 0.56	0 (0.06)

*t* is the test statistic. Roll (°) gives the average roll value. Pitch (°) gives the average pitch value. *p* value of the test, returned as a scalar value in the range [0,1], presents the probability of accepting (*p* > 0.05, *h* = 0) or rejecting (*p* ≤ 0.05, *h* = 1) the null hypothesis

**Fig. 12:**
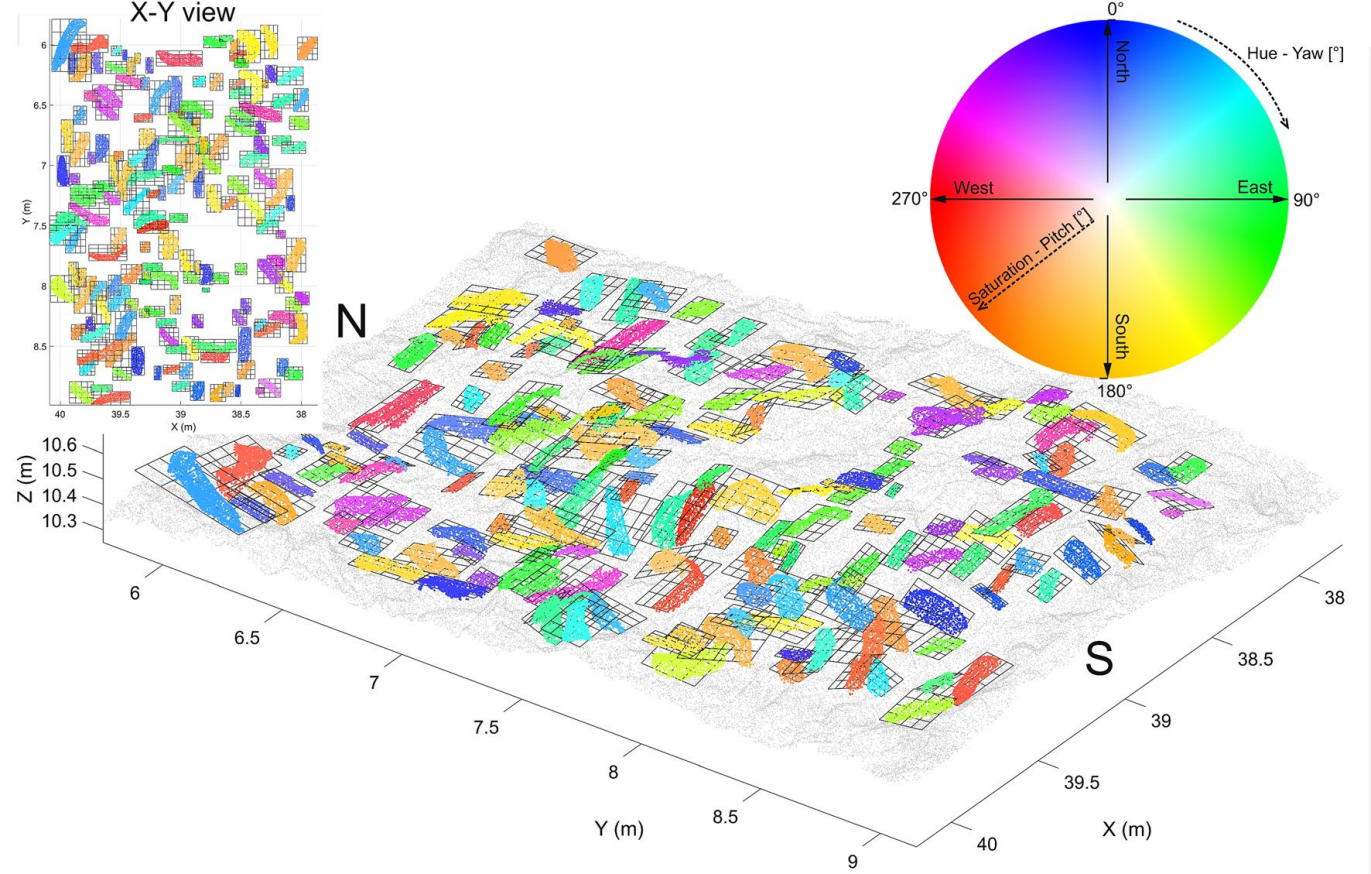
Orientation map of one tile size 3 × 2 m, comprising 177 shells. Color wheel coding depends on the angles. Hue encodes orientation angle yaw and saturation encodes pitch angle, while roll angle is not visualized. The color is darker on the places, where the pitch (tilt) is larger or lighter if the shell is approximately horizontal

An intuitive option to visualize such 3D-orientation data is applying the color codes of a color wheel (Figs. 5, 12). Hue encodes the orientation angle yaw and saturation encodes pitch, while roll is not visualized. The color is darker on those places, where the pitch (tilt) is larger, and lighter if the shell is approximately horizontal. The color wheel ranges from 0°–360°. A yaw angle $\gamma$ ranging from 0° to 90° is coded with blue to green colors (Fig. 12). A yaw angle $\gamma$ between 90° and 180° is visualized in green-to-yellow color; from 180° to 270°, the color code stretches from yellow to red, and for yaw from 270° to 360°, the color will range from red to blue. The color coded, automatically derived results for all 13 tiles with manually defined shells is shown in Fig. 3. Illustration of each angle in a separate map with its own color bar is shown in Fig. 10.

Student *t* test values are given in Table 1. Using the one-sample *t* test, it is evaluated if mean roll and pitch for each tile are equal to the overall mean. *p* value of the test, returned as a scalar value in the range [0,1], presents the probability of accepting or rejecting the null hypothesis. The null hypothesis (*h* = 0) is accepted if the p is larger than 0.05. This is the case for 10 tiles for the roll angle and 11 tiles for the pitch angle. This points that there is no significant mean difference between the compared tiles, while for tiles 3806, 3812, and 3821 (see tile map on Fig. 3) of the roll angle, the null hypothesis is rejected (*h* = 1). The same is observed for the tiles 3812 and 4212 of the pitch angle. As the mean roll (0.74°) and the mean pitch (1.11°) values do not deviate significantly from 0°, the null hypothesis has to be rejected at the confidence level 5%.

The Kolmogorov–Smirnov test is used to study if an empirical distribution follows an expected (i.e., predefined) distribution in the interval [0°, 360°]. The cumulative distribution is generated from ascending sorted yaw angles (values from 0 to 360). The cumulative distribution as a curve which is always increasing by 1/1904, for each yaw value, and the differences (*Dn*) between the values of the cumulative distribution function (cdf) and the reference. If the sample data are uniformly distributed, these differences should be zero. *Dn* is the largest value and in our case is 0.05. If the data are uniformly distributed, then the critical value *Dn,α* should be larger than *Dn*. From the Kolmogorov–Smirnov table, it is obvious that *Dn,α* = *D*_1904,0.05_ = 1.36/sqrt(1904) = 0.031. Since *Dn* = 0.05 > 0.031 = *Dn*,α, we conclude that the data do not follow a uniform distribution.

**Fig. 13:**
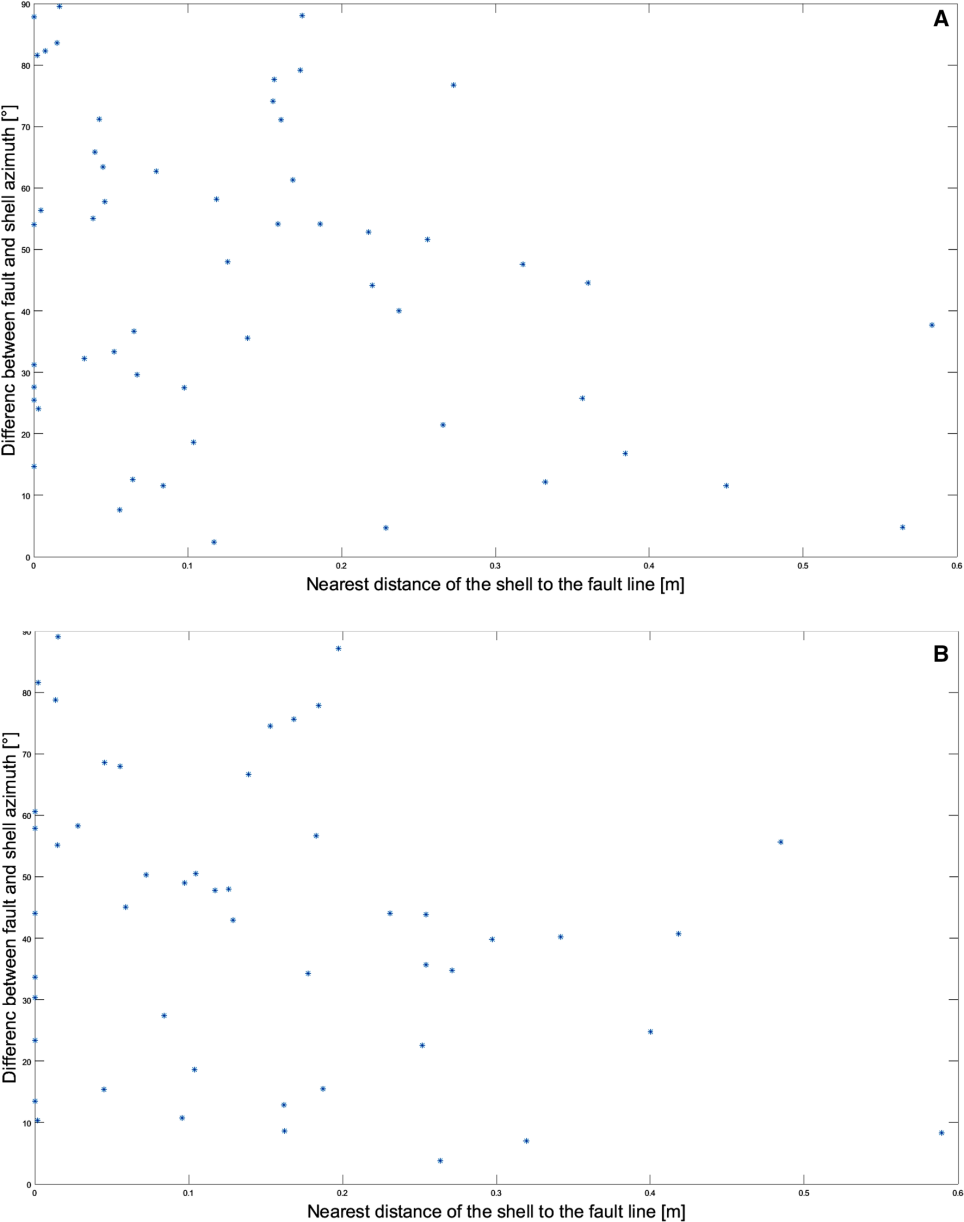
**a** Shells with pitch angle tilt above 18.4°; **b** shells with roll angle tilt above 31.5°

To quantify the effect of post-sedimentary displacement by faults on shell orientation, we analyzed the relation between strongly tilted shells close to the fault lines (Fig. 7): shells shown in blue present roll angle above 31.5° and yellow shells present pitch angle tilt above 18.4°. We show the difference between fault and shell azimuth versus nearest distance of the shell to the fault line for the shells with pitch angle tilt above 18.4° (Fig. 13a) and for the shells with roll angle tilt above 31.5° (Fig. 13b). Figures 7 and 13 illustrate the individual shells on the reef bed within the multi-buffers surrounding the fault lines. They are in close distance to the fault lines or on it. The close distance is described via multi-buffer distances: blue: 0.1 m, pink: 0.2 m, and green: 0.3 m (Figs. 6, 7). The accumulative values (buffer wise) are displayed in Table 2. Quantification parameters were the nearest distance of individual shells from the fault line and the shell angles (*φ* > 18.4° or *ρ* > 31.5°). Table 2 shows that the accumulative number of the oyster shells with outlying orientation pitch angles has differences up to 2.2% points compared to that of all the shells in the tile. For outlying roll angles, the corresponding accumulative distance ratios vary more, being up to 10.0% points for 0.10 m buffer and then decreasing to 2.2% points for the 0.30 m buffer. In terms of oyster shell numbers, these differences are one shell for the pitch angle and five shells for the roll angle. These numbers indicate that the fault line vicinity does not seem to have an effect to the high outlying pitch angle values. A similar assumption can be made about the shells with high outlying roll angle values even while they are not as strongly correlated. This would be in line with Harzhauser et al. (2015), because tectonic activity postdates sedimentation distinctly.

**Table 2:** Buffer zones: accumulative ratio of individual oyster shells within the buffer zones of the nearest fault line (see also Fig. 6)

Buffer distance (m)	Oyster shells with pitch angle *φ* > 18.4° (*N* = 47)	Oyster shells with roll angle *ρ* > 31.5° (*N* = 58)	All shells (1904)—outliers (99)
0.01	20.4	17.7	21.4
0.05	34.4	28.1	36.9
0.10	48.4	40.6	50.6
0.20	73.1	67.7	72.5
0.30	88.2	84.4	86.6

Please note that the two first buffers are not visualized on Fig. 6

## Discussion

**Fig. 14:**
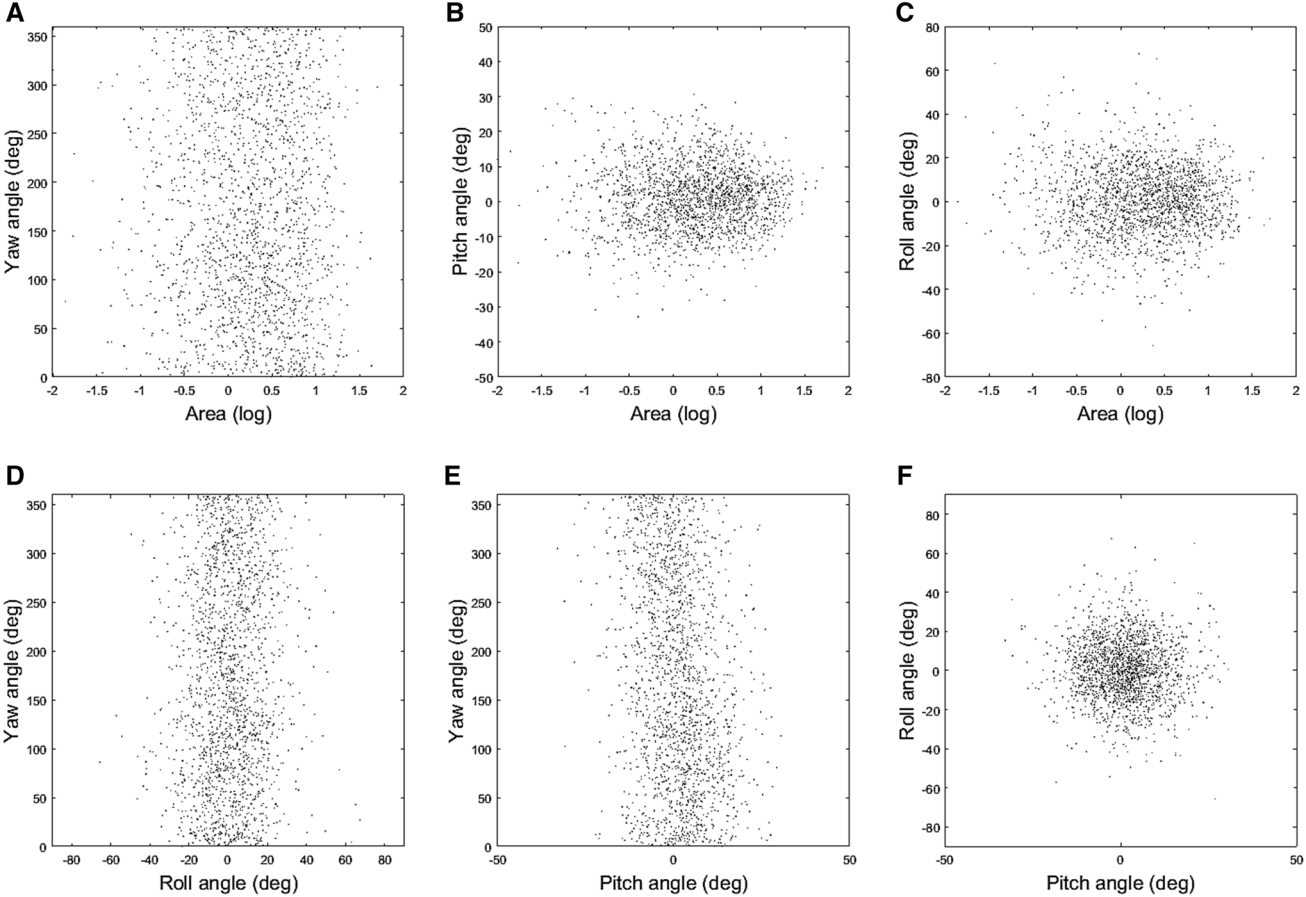
Cross plots showing correlation between area and yaw-, pitch-, and roll angles (**a**–**c**) and correlations between the individual angles (**d**–**f**)

A smaller part of the data set used herein was analyzed also by Harzhauser et al. (2015), who tried to decipher the complex taphonomic history of the shell bed. There, the authors hypothesized that either a tsunami or an exceptional storm formed the event layer and further suspected that the hydrological regime might still be mirrored in shell orientations. They quantified shell orientations of 527 manually delineated specimens using the 2D centreline of each object. The centreline was automatically derived from oyster boundaries and is defined as an imaginary curved line spanning the maximum length of the shell using the method from Djuricic et al. (2016c). The study detected a weak preference for WSW–ENE orientations, but concluded that potential original patterns were biased by post-event processes. In this experiment, our extended data set confirms the observations made by Harzhauser et al. (2015) for oyster shell yaw angles. In addition, we include the additional information about the shell’s start and end direction which indicates a preponderance rather from West to East than from East to West. This pattern, however, is statistically insignificant in tests for preferred directions. Nevertheless, a significant orientation pattern might be masked by lumping of shells of different size classes. Shells of different sizes differ in weight and will react differently in a given flow regime. Therefore, shell-surface area data from Harzhauser et al. (2016) for all 1904 shells were used to detect potential correlations of size with yaw, pitch, and roll angles (Fig. 14a–c). Again, no correlation can be detected in the cross plots and statistical tests reject any significant correlation.

The 2D approach of Harzhauser et al. (2015) failed to detect patterns associated with roll and pitch angles. Both might reveal imbrication, which is an important feature of storm-related surge deposits (Spiske and Jaffe 2009) and tsunami-generated deposits (Bahlburg and Spiske 2012). If strong surge would have caused imbrication, the shells would have been stacked parallel to the longer axes, which would be reflected in the roll angels. Indeed, the roll angles display two times larger variation compared to the pitch values (Fig. 13), but lack a persistent pattern. Thus, the observed variation results from random stacking and partial overlaying of shells within the densely packed shell bed, whereas imbrication can be ruled out. In addition, the generally low pitch angles document that the shells are laying more or less subparallel to the bedding plane. No chaotic orientation in the sediment can be detected, which could be indicative for gravity and debris flows (Kidwell and Bosence 1991; Hladil et al. 1996). This strongly suggests that the shells were part of the sea bottom surface rather than having been admixed in the surrounding sand by mass transport.

All these data support a former hypothesis by Harzhauser et al. (2015), who suggested that the shells were exposed on the seafloor after the storm or tsunami event for several years. During this post-event phase, all potential orientation would have been strongly biased leading to the observed random distribution.

The Student *t* test statistic says that the roll value is significantly different from zero (Table 1). This can be supported by a calculation of the effect. The mean roll is 0.74°. For a shell width of approximately 10 cm, this means a height difference of 1 mm from left side to right side. Thus, statistical analysis suggests a slight preference, but it is on the border of the measurement and outline accuracy. Concerning the pitch angle, the mean value is a bit larger (1.11°), and the length of the shells is also larger than the width. The average length of the shells is 24 cm, which makes a height effect of 5 mm. This suggests that the hinge area, where is a ligament, is heavier than the muscle area.

Assuming the absence of imbrication, it does not make a difference if a pitch or roll angle is positive or negative. Recalculating the pitch angles all as positive results in a mean of 7.1° (std. 5.7°), with the 25 and 75 percentiles ranging from 2.7° to 10.1° (Table 3). For roll angles, the mean is 11.9° (std. 9.8°) with the 25 and 75 percentiles ranging from 4.6° to 16.6°. These ranges are explained by a large number of shells lying more or less subparallel in respect to the bedding plane and a contribution by moderately inclined specimens, due to partial overlap with neighboring shells. Outliers are thus characterized by 58 shells (3.1% of all shells) having pitch angles with over 18.4°, which is two times standard deviation compared to the mean. Respectively, 47 shells (2.5%) have roll angles with over two times standard deviation (31.5°) compared to the mean. Total number of outlier shells (pitch or roll) is 99 (5.2%). Shared number of outlier shells (pitch and roll) is 6. Hence, only 6.1% of outlier shells have both angles in outlying region; therefore, pitch and roll are likely not linked. Figures 7 and 13 illustrate those shells on the reef bed and show location depending on the position of fault lines. They are lying in close distance to the fault lines or on it (see values in Table 2 and Supplementary Table 4).

The 2D approach of Harzhauser et al. (2015) was unable to detect the impact of local faults, which are well visible in the digital surface model (Figs. 6, 7). The shell bed is cut by a set of subparallel, NNW–SSE trending, and steeply dipping normal faults, accompanied by a subordinate NE–SW trending set. The ca. 0.5–10 m-long fault traces can be identified in the outcrop and on the DSM, vertically displacing the oyster-bearing sandy layer by a maximum of 30 cm. In addition, individual oyster shells are frequently fractured and displaced along the faults. To quantify the influence of displacement by faults on the orientation of the shells, roll and pitch angles of shells along the fault lines were checked by applying an accumulative, bufferwise approach. Buffers of 0.01, 0.05 m, and three buffers of 0.1 m width were defined around the reference fault line (Fig. 6). Although the multi-buffers documented the strongest tilt of the shells close to the fault line, over 84% for the both inspected angles, also the majority of all other shells (86.6%) are within buffer zones up to 0.3 m. However, it reveals that these shells must be excluded from analysis of primary orientation patterns.

**Table 3:** Overview of the results regarding yaw, pitch, and roll angle values

Rotation angles	Yaw (*γ*°)	Pitch (*φ*°)	Roll (*ρ*°)
Total number (tn)	1904 shells		
Mean	168.8	1.11	0.74
Standard deviation	104	9.0	15.4
Angle interval	(0 360)	(− 90 + 90)	(− 90 + 90)
Percentile 25	/	2.7	4.6
Percentile 75	/	10.1	16.6
Outliers*	/	3.1%(tn)	2.5%(tn)

*Note that outlier is counted if φ > 18.4° or ρ > 31.5°

## Conclusion

The aim of this paper is to demonstrate an interdisciplinary method to calculate 3D orientations of elongated objects in Earth Science such as fossilized oyster shells from a densely packed Miocene shell bed based on approaches developed in photogrammetry. We estimated the individual shell orientations with respect to the superimposed “horizontal geological” coordinate system named LACS (Djuricic et al. 2016a). To set the orientation of an oyster shell, the reference coordinate system was used as well as the individual shell coordinate system. The orientation was specified by aerial photogrammetry principle angles implemented to determine rotations of complex objects. Those principal rotations are known as yaw, pitch, and roll angle and they are used to describe the orientation of a 3D oyster in any orientation. Terrestrial laser scanning and the resulting high-resolution 3D-point cloud provide the base for non-destructive analyses, which is a major advantage in protected natural heritage sites and in areas, which are difficult to access. A high level of objectivity is warranted by the reproducibility of any data processing and subsequent analyses.

This study, focusing on a Miocene oyster biostrome, summarizes the technical and methodological frame to acquire the data and highlights the high potential for paleoecological, sedimentological, and taphonomic analyses. Although designed as methodological paper, our data confirm the previous taphonomic interpretations of Harzhauser et al. (2015). The oyster shells in the event-bed lack a significant orientation of the yaw angles. This suggests a complete loss of potential current patterns related to the storm or tsunami event that formed the shell bed during a longer phase of exposure. Based on the large data set on the orientation of 1904 shells, the earlier reported weak preponderance of W–E orientations was shown to be statistically insignificant. The random distribution of the pitch and roll angles clearly excludes any imbrication fabrics, which could have been related to tsunami- or storm-generated surge currents. 6.1% of examined oyster shells were labelled as outliers based on their high pitch and roll angles. Some of the outlier values can be explained by stacking of neighboring or partly overlaying shells in the densely packed shell bed. While the majority of the shells with outlying pitch or roll angle values were found within 0.30 m from the nearest fault line, their ratio did not show statistically significant differences compared to that of all shells. Therefore, no clear relationship between fault lines and strong rotation angles can be documented. Although many shells along the faults were broken and displaced along the strike-slip faults, post-sedimentary vertical displacement by faults cannot be directly related to shell orientation. The oyster reference database allows testing and analyzing of possible additional relationships between shell anomalies and outlying tilt angle values.

The method can be applied in comparable scientific fields to any kind of fossilized bed or other geological elongated objects to examine their spatial orientation to conclude on their environmental deposition patterns. Oyster reef data set (in the form of digital surface model, corresponding hill shade data, and orthophoto data) can be accessed via PANGAEA at 10.1594/PANGAEA.863615 (see Djuricic et al. 2016b).

## Electronic supplementary material

Below is the link to the electronic supplementary material.

**Appendix** This paper contains an appendix, which includes determination of the individual shell coordinate system (ISCS), the rotation matrix (A) of the axes X_ISCS_, Y_ISCS_, and Z_ISCS,_ and the angles yaw - $\varvec{\gamma }$, pitch – $\varvec{\phi }$, and roll -$\varvec{\rho }.$ (DOCX 17 KB)

**Table 4**: Data set of 1904 shells including attributes: shell ID, tile, taxon, visible side, nearest fault line ID, nearest distance, azimuth of the fault line, yaw-, pitch-, roll-angle, length, and area. (XLSX 223 KB)

## Footnotes

1 Note that *ρ* is counted mathematically positive, contrary to *γ* and *φ*.
