# Supplementary material for: Automatic determination of 3D orientations of fossilized oyster shells from a densely packed Miocene shell bed
Source: Int J Earth Sci. 2018 Feb 23;107(6):2125–42. doi: 10.1007/s00531-018-1591-0 (PMC6096536; doi:10.1007/s00531-018-1591-0)
Supplement: Supplementary file 1 — Appendix This paper contains an appendix, which includes determination of the individual shell coordinate system (ISCS), the rotation matrix (A) of the axes XISCS, YISCS, and ZISCS, and the angles yaw - $\varvec{\gamma }$γ, pitch – $\varvec{\phi }$ϕ, and roll -$\varvec{\rho }.$ρ. (DOCX 17 KB) [file 531_2018_1591_MOESM1_ESM.docx]

**APPENDIX**

The matrix (A) of the axes X_ISCS_, Y_ISCS_, and Z_ISCS_ is:

$$A=\left( \upsilon_{1}, \upsilon_{2}, \upsilon_{3} \right)$$

Here $\upsilon_{1}, \upsilon_{2}, \upsilon_{3}$ are the eigenvectors for the sorted eigenvalues λ_1_ > λ_2_ >λ_3_ of the second centralized moments of the 3D points on one shell. In order to uniquely determine angles from the rotation matrix $A$ the orientation of the eigenvectors have to be checked by the following criteria:

i) The determinant of A must be positive, i.e. det(A) = +1. If determinant of A is negative, i.e. det(A) = -1, then A is redefined as A: = -A.

ii) If υ_3_ is pointing downwards, i.e. υ_3, z_ < 0, then redefine A accordingly:

$$A=\left( \upsilon_{1}, -\upsilon_{2},-\upsilon_{3} \right).$$

iii) Without further context, the orientation of υ_1_ cannot be determined and it should be set to point always (e.g.) eastwards. The yaw angle γ is then restricted to [0°,180°]. If, on the other hand, context knowledge allows determining the orientation of υ_1_, then γ can be determined in [0°,360°]. This introduces two paths in the method:

iii-a) No orientation, υ_1_ points eastwards: Should υ_1, x_ < 0, then A is redefined to:

$$A=\left( -\upsilon_{1},-\upsilon_{2}, \upsilon_{3} \right).$$

iii-b) Orientation given by context knowledge: Here, the hinge position (start of the shell) or muscle position (approximately the end of the shell) can be added to the data as a descriptive attribute, using, e.g., the oyster center line (Djuricic et al., 2016). Therefore, the direction of the length axis can be given in a range of 0°–360°. Then verify:

$$\upsilon_{1, 2D}^{T}\cdot( {\binom{x}{y}}_{muscle}- {\binom{x}{y}}_{hinge})>0.$$

The index *2D* refers to the vector composed only of the *x* and *y* coordinate. If the verification provided a value below 0, then redefine

$$A=\left( -\upsilon_{1},-\upsilon_{2},\upsilon_{3} \right).$$

The matrix of the axes (A) is after these transformations a right handed coordinate system, named the individual shell coordinate system (ISCS, Fig. 3). The first axis, i.e. the first column vector, is pointing from hinge to muscle, the third axis, i.e. the third column vector, is pointing upwards.

The rotation matrix

$$\left( {cos-\left( 90^{\circ}+\gamma\right) \atop\begin{aligned} sin-\left( 90^{\circ}+\gamma\right) \\ 0 \end{aligned}}{-sin-\left( 90^{\circ}+\gamma\right) \atop\begin{aligned} cos-\left( 90^{\circ}+\gamma\right) \\ 0 \end{aligned}}{0 \atop\begin{aligned} 0 \\ 1 \end{aligned}} \right)\left( {cos-\varphi\atop\begin{aligned} 0 \\ -sin-\varphi\end{aligned}}{0 \atop\begin{aligned} 1 \\ 0 \end{aligned}}{sin-\varphi\atop\begin{aligned} 0 \\ cos-\varphi\end{aligned}} \right)\left( {1 \atop\begin{aligned} 0 \\ 0 \end{aligned}}{0 \atop\begin{aligned} cos\rho\\ sin\rho\end{aligned}}{0 \atop\begin{aligned} -sin\rho\\ cos\rho\end{aligned}} \right)$$

is simplified to:

$$\left( {cos\left( 90^{\circ}+\gamma\right) \atop\begin{aligned} -sin\left( 90^{\circ}+\gamma\right) \\ 0 \end{aligned}}{-sin\left( 90^{\circ}+\gamma\right) \atop\begin{aligned} cos\left( 90^{\circ}+\gamma\right) \\ 0 \end{aligned}}{0 \atop\begin{aligned} 0 \\ 1 \end{aligned}} \right)\left( {cos\varphi\atop\begin{aligned} 0 \\ sin\varphi\end{aligned}}{0 \atop\begin{aligned} 1 \\ 0 \end{aligned}}{-sin\varphi\atop\begin{aligned} 0 \\ cos\varphi\end{aligned}} \right)\left( {1 \atop\begin{aligned} 0 \\ 0 \end{aligned}}{0 \atop\begin{aligned} cos\rho\\ sin\rho\end{aligned}} {0 \atop\begin{aligned} -sin\rho\\ cos\rho\end{aligned}} \right)=$$

$$\left( {-sin\gamma\atop\begin{aligned} -cos\gamma\\ 0 \end{aligned}}{cos\gamma\atop\begin{aligned} -sin\gamma\\ 0 \end{aligned}}{0 \atop\begin{aligned} 0 \\ 1 \end{aligned}} \right)\left( {cos\varphi\atop\begin{aligned} 0 \\ sin\varphi\end{aligned}}{-sin\varphi sin\rho\atop\begin{aligned} cos\rho\\ cos\varphi sin\rho\end{aligned}}{-sin\varphi cos\rho\atop\begin{aligned} -sin\rho\\ cos\varphi cos\rho\end{aligned}} \right)=$$

$$\left( {-sin\gamma cos\varphi\atop\begin{aligned} -cos\gamma cos\varphi\\ sin\varphi\end{aligned}}{sin\gamma sin\varphi sin\rho+cos\gamma cos\rho\atop\begin{aligned} cos\gamma sin\varphi sin\rho-sin\gamma cos\rho\\ cos\varphi sin\rho\end{aligned}}{sin\gamma sin\varphi cos\rho-cos\gamma sin\rho\atop\begin{aligned} cos\gamma sin\varphi cos\rho+sin\gamma sin\rho\\ cos\varphi cos\rho\end{aligned}} \right)= \left( {r_{1,1} \atop\begin{aligned} r_{2,1} \\ r_{3,1} \end{aligned}}{r_{1,2} \atop\begin{aligned} r_{2,2} \\ r_{3,2} \end{aligned}}{r_{1,3} \atop\begin{aligned} r_{2,3} \\ r_{3,3} \end{aligned}} \right)$$

Using the matrix elements$r_{1,1}$, $r_{2,1}$ and $r_{3,1}$ in further computation, the angles yaw - $\gamma$, pitch - $\varphi$ and roll - $\rho$ can be computed.

$$tan\gamma= \frac{r_{1,1}}{r_{2,1}}$$

When $\gamma$ is in a range of 0°-180° (path iii-a), if the inverse function of tan provides a value below 0°, then 180° is added to $\gamma.$

When $\gamma$ is in a range of 0°-360°(path iii-b), the function “tan2”, i.e. the function considering the quadrants and the signs of counter and nominator, has to be used.

$$\sin\varphi= r_{3,1}$$

The inverse sin function provides pitch angle φ values between -90° and +90°.

$$sin\rho= \frac{r_{3,2}}{cos\varphi}$$

Roll angle ρ is in the range between -90° to +90°.
